# An exercise and patient education intervention to reduce pain and physical limitations in adults with acetabular dysplasia: study protocol for a process evaluation integrated within a randomised controlled trial (the MovetheHip trial)

**DOI:** 10.1186/s13063-024-08262-y

**Published:** 2024-06-24

**Authors:** Julie S. Jacobsen, Rhiannon Evans, Kelly Morgan, Kristian Thorborg, Lisa G. Oestergaard, Dorthe Sørensen

**Affiliations:** 1 Research Centre for Rehabilitation, VIA University College, Hedeager 2, Aarhus N, 8200 Denmark; 2 Research Unit for General Practice, Bartholins Allé 2, 8000 Aarhus, Denmark; 3 Centre for Development, Evaluation, Complexity and Implementation in Public Health Improvement (DECIPHer), School of Social Sciences, SPARK, Cardiff University, Maindy Road, Cardiff, UK; 4 Department of Orthopaedic Surgery, Sports Orthopaedic Research Center-Copenhagen (SORC-C), Copenhagen University Hospital, Amager-Hvidovre, Kettegård Alle 30, 2650 Hvidovre, Denmark; 5 Department of Physical and Occupational Therapy, Physical Medicine and Rehabilitation Research-Copenhagen (PMR-C), Copenhagen University Hospital, Amager-Hvidovre, Kettegård Alle 30, 2650 Hvidovre, Denmark; 6 DEFACTUM, Central Denmark Region, P.P. Ørums Gade 11, 8000 Aarhus C, Denmark; 7 Department of Public Health, Aarhus University, Bartholins Allé 2, 8000 Aarhus, Denmark

**Keywords:** Hip pain, Training, Self-management, Mechanisms of change, Acceptability, Implementation, Context

## Abstract

**Background:**

The Movethehip trial investigates the effectiveness of an exercise and patient education intervention for adults with acetabular dysplasia. The intervention involves eight tailored one-to-one sessions with trained providers who employ supportive feedback tools. The present protocol reports a planned process evaluation, which aims to determine how the intervention functions by examining the implementation of the intervention (process, dose and reach), its acceptability, mechanisms of change and the influence of contextual factors.

**Methods:**

Two hundred trial participants aged 18–50 years will be recruited from a University Hospital in Denmark and randomised to the intervention or control group. Approximately ten providers will deliver the intervention. The process evaluation adopts a concurrent mixed-methods design. The implementation will be assessed using self-report questionnaires (at baseline and 6-month follow-up), training records and semi-structured focus group interviews with intervention providers (*n* = 10) and healthcare managers (*n* = 4–6). The mechanisms of change will be explored through semi-structured one-to-one interviews (at baseline and 6-month follow-up) with 15–20 purposefully sampled participants and by measuring changes in health outcomes (self-reported pain, physical functioning and quality of life completed at baseline and at 3- and 6-month follow-up). Additionally, change will be measured through an explorative examination of associations between dose and change in health outcomes, applying simple linear regression models. The acceptability of the intervention and the influence of contextual factors will be explored through one-to-one participant interviews and focus group interviews with 4–6 healthcare managers. The interviews will focus on expectations, experiences, events, personal understandings and interaction with interpersonal and organisational aspects. Interview data will be analysed using theoretical thematic analyses, and findings will be merged with quantitative data and reported jointly on a theme-by-theme basis.

**Discussion:**

The process evaluation conducted as part of the MovetheHip trial will illuminate how the intervention functions, and if the intervention is proven effective, the findings of the evaluation will contribute to pinpoint how the intervention may be optimised to facilitate future up-scaling and implementation.

**Trial registration:**

The MovetheHip protocol was approved by the Committee on Health Research Ethics in the Central Denmark Region. ClinicalTrials, NCT04795843. Registered on 20 March 2021.

**Supplementary Information:**

The online version contains supplementary material available at 10.1186/s13063-024-08262-y.

## Background

Acetabular dysplasia is a condition characterised by reduced acetabular coverage of the femoral head [1]. It is most commonly seen in young-to-middle-aged persons [2]. Pain and physical limitations imposed by acetabular dysplasia may be improved surgically by periacetabular osteotomy (PAO) [2–4]. However, several barriers to surgery have been reported [5–7]. These barriers include having a Body Mass Index above 25, being above 45 years or having hip osteoarthritis. Thus, a PAO may not be offered as a treatment option to persons with these characteristics due to a heightened risk of adverse outcomes [5–7]. In addition, some eligible persons may not be willing to undergo surgery. Both candidates for surgery and those who do not undergo surgery may experience pain and detrimental effects on their physical and mental well-being [8, 9]. Such effects may include feeling controlled by their hip pain, being limited in their participation in social and physical activities of everyday life, and worrying about their future [8].

In persons not undergoing surgery, exercise may potentially be an alternative to reduce pain and improve physical functioning [10–12]. However, the evidence base for exercise interventions aiming to treat symptoms in acetabular dysplasia is weak. Only small pilot and feasibility studies have been published [10–12], and none of these studies focused on people not undergoing surgery [10–12]. Furthermore, study limitations were described. These limitations include insufficient intervention description and low recruitment or exercise adherence. Furthermore, all studies failed to monitor motivation for exercise and physical activity in everyday life [10–12]. However, participants’ motivation for exercise and physical activity is critical [13], especially in persons with acetabular dysplasia who experience a lack of control and are limited in relation to social and physical activities [8]. A range of theories have been developed to understand the complexity of motivation, including self-determination theory [14, 15] and motivational interviewing [16]. Evidence supports that interventions based on these two theories effectively promote physical activity [17–19].

We developed an intervention that integrates motivation for exercise and physical activity for adults with acetabular dysplasia who are not undergoing surgery. The intervention was prepared in accordance with Medical Research Council (MRC) guidance on the development and evaluation of complex interventions [20]. We conducted a feasibility study on this intervention [9]. This feasibility study confirmed that it was feasible to conduct a full-scale randomised controlled trial (RCT) with an integrated process and economic evaluation entitled the MovetheHip trial. The protocol for the MovetheHip trial has been reported elsewhere [21]. The present protocol reports the planned process evaluation following the MRC guidance on process evaluations [22, 23].

## Programme theory

### Mechanisms of change

The MovetheHip intervention aims to reduce pain and physical limitations by enhancing hip muscle strength through physical exercises and physical activity, while also assisting participants in managing their condition in everyday life.

**Fig. 1:**
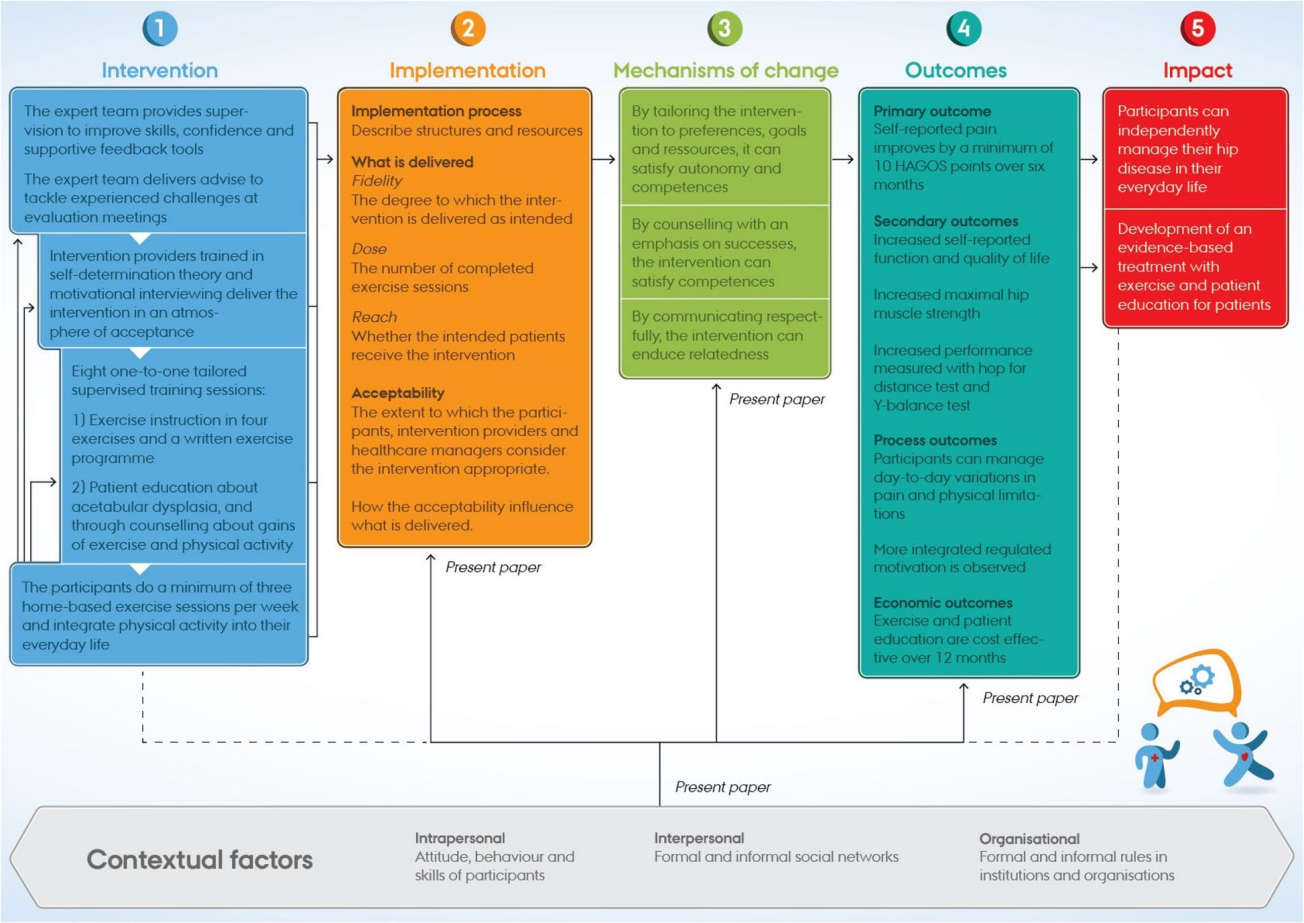
MovetheHip logic model with implementation, acceptability, mechanisms of change and contextual factors

Motivation for exercise and physical activity is critical, and the participants are considered more vulnerable than background populations without pain because they have to sustain motivation for exercising despite experiencing pain and discomfort [13]. Therefore, the mechanisms of change are rooted in self-determination theory and motivational interviewing. Furthermore, being rooted in the self-determination theory, the intervention incorporates the psychological determinants of autonomy, competence and relatedness [14, 15]. By targeting these determinants, we hypothesise that the intervention will shift behaviour from motivation by external control towards more integrated regulated motivation [15, 24] leading to integration of exercises and physical activity into participants’ everyday lives, thereby increasing their physical and mental well-being [13–15] (Fig. 1).

### MovetheHip intervention components

The intervention is delivered by providers who are trained in self-determination theory and motivational interviewing. It involves two components: an exercise programme and patient education.

#### MovetheHip exercise programme

Eight supervised physical one-to-one sessions will be delivered in the course of 6 months [9, 21], and each session takes 30–45 min (time is recorded). In the supervised sessions, an intervention provider will guide the participant through four exercises [9, 21]. These exercises are (1) a supine plank exercise, (2) a side-lying plank exercise, (3) a squat exercise and (4) a one-leg stability exercise. Each exercise may be performed at one of three predefined difficulty levels, with exercise repetitions ranging from 5 to 20. All participants will start at the lowest difficulty level (C) and move to higher levels (B and A) over time based on their Borg CR10 Scale score [25] and individual preferences, goals and resources. Similarly, individual adjustments will be made with respect to exercise repetitions. The participants will be encouraged to perform a minimum of three weekly training sessions at home. Each session takes approximately 20–30 min.

#### MovetheHip patient education

The participants will also receive one-to-one verbal patient education at each supervised session based on their individual needs [9, 21]. The education will focus on acetabular dysplasia, the rationale and importance of being physically active and exercising regularly. Additional educational topics include tissue tolerance and pain mechanisms, gains from exercise and the association between being overweight and experiencing pain.

Part of the patient education will include encouraging participants to fit exercises and physical activity into their everyday lives. They will receive support to manage any experienced barriers between the supervised sessions and be encouraged to adjust the exercises to their level of functioning, time available and personal resources. The counselling provided will be non-judgemental, respectful and empathic, aiming to enhance the participants’ motivation to integrate exercises and physical activity into their everyday lives [13, 16].

### MovetheHip implementation strategy

The implementation strategy is empirical, based on observations made in an orthopaedic outpatient clinic at a University Hospital in Denmark and in public and private clinics in Denmark.

#### Recruitment of participants

Persons with symptoms and a radiograph consistent with acetabular dysplasia will be referred from general practice to an outpatient clinic at a Danish University Hospital. At the clinic, an orthopaedic surgeon will provide a diagnosis and, together with the person, decide on the necessity of surgery. As part of this procedure, the participants will be recruited by orthopaedic surgeons at the outpatient clinic [9, 21]. Surgeons were involved in the planning of recruitment procedures and the drafting of written recruitment material. The recruitment material includes a short checklist of procedures, contact information for the principal investigator (first author), a screening form and written material for the participants. The material will be placed in each clinic room and will remain accessible anytime for surgeons. The principal investigator will hold regular meetings with the surgeons, discussing the eligibility of individual participants.

#### Identification of intervention providers

The intervention providers will be physiotherapy students from a university college in Denmark [21]. Written and oral advertisement materials are shared with second-year students, and selected students will serve as intervention providers until they graduate. Three months before graduation, new students will gradually replace former intervention providers over a 2-month period until all participants have completed their intervention period.

#### Training of intervention providers

Intervention providers will deliver the intervention under the supervision of an expert team of physiotherapists (a senior lecturer, the first and fourth authors) [9, 21]. Each provider will receive two 2-h training workshops and 1 h of one-to-one supervision per participant to teach providers how to deliver the intervention and counsel participants in line with self-determination theory and motivational interviewing. The providers will be trained to spot any signs of barriers and lack of preparedness for exercise and for engaging in physical activity and will learn how to respond to distress or need for support in a manner that satisfies the need for autonomy, competence and relatedness. During the intervention period, the students and the expert team will attend regular evaluation meetings to ensure that the intervention providers feel prepared to deliver an individually tailored intervention.

### The influence of the context

The mechanisms of change will be underpinned by an ecological view of change. We hypothesise that characteristics of the context (i.e. personal, interpersonal and organisational factors) will interact with the fulfilment of the need for autonomy, competence and relatedness, ultimately influencing the quality of expected and experienced behaviour [26].

## Process evaluation aim

The present protocol reports the planned process evaluation, which aims to determine how the intervention functions by examining the following three domains: (I) Implementation of the intervention (process, dose and reach), (II) Acceptability of the intervention, (III) Mechanisms of change and the influence of contextual factors across the three domains.

### Process evaluation research questions

The domains of the process evaluation will be examined by answering the following overarching research questions (further details are provided in Table 1):

1. Is the intervention delivered with fidelity, and how do contextual characteristics structure fidelity?
2. Is the intervention acceptable to the participants, the intervention providers and the healthcare managers, and how do contextual factors interact with acceptability?
3. Does the intervention work in accordance with the proposed mechanisms of change, and how do contextual characteristics influence these mechanisms?

**Table 1:** The process evaluation domains in relation to research questions, data sources and procedures

Domains	Research questions	Data sources	Procedures	When	Informants
**Implementation**	How do observations and experiences (context) relate to tailoring to each participant, and how do they relate to intervention fidelity?	Focus group interviews	Interview led by the last author (evaluation meetings)	Throughout trial	Intervention providers (*n* ≈ 10) and expert team^c^ (*n* ≈ 3)
		Online survey	Self-report: fidelity as the extent which intervention was delivered, 100 mm VAS	Six months	Intervention providers (*n* ≈ 10)
**Implementation**	Do baseline characteristics differ between participants who received high and low intervention doses and in participants and non-participants (intervention reach)?	Online record	Self-report	Baseline	Participants (*n* ≈ 100)
		Online record	Clinician registration: radiographical variables	Baseline	Participants (*n* ≈ 100)
		Online rating scale^b^	Self-report: exercise dose	Six months	Participants (*n* ≈ 100)
**Implementation** **(explorative)**	How does dose relate to changes in health outcomes?	Online PROM^a^	Self-report: pain, physical functioning and QOL	Baseline, 3 and 6 months	Participants (*n* ≈ 100)
		Online rating scale^b^	Self-report: exercise dose	Six months	Participants (*n* ≈ 100)
**Acceptability**	How do contextual factors relate to expectations and behavioural experiences?	1:1 semi-structured interviews	Interview led by the first author	Baseline and 6 months	Participants (*n* ≈ 15–20)
**Acceptability**	How prepared do providers feel to deliver the intervention?	Focus group interviews	Interview led by the last author (evaluation meetings)	Throughout trial	Intervention providers (*n* ≈ 10) and expert team^c^ (*n* ≈ 3)
**Acceptability**	How do contextual factors relate to perceptions of a forthcoming implementation?	Focus group interview	Interview led by the last author	When 50% of the participants have been recruited	Healthcare managers and practice consultants (*n* ≈ 4–6)
**Acceptability** **(explorative)**	Does the reason for deselecting surgery moderate dose?	Online rating scale^b^	Self-report: exercise dose	Six months	Participants (*n* ≈ 100)
		Online record	Clinician registration: reason to deselect surgery	Baseline	Participants (*n* ≈ 100)
**Mechanisms of change**	Does intervention functioning relate to a change in health outcomes?	1:1 semi-structured interviews	Interview led by the first author	Baseline and 6 months	Participants (*n* ≈ 15–20)
		Online PROM^1^	Self-report: pain, physical functioning and QOL	Baseline, 3 and 6 months	Participants (*n* ≈ 100)
**Mechanisms of change**	How do contextual factors structure intervention functioning?	1:1 semi-structured interviews	Interview led by the first author	Baseline and 6 months	Participants (*n* ≈ 15–20)

*PROM* Patient-reported outcome measure, *QOL* Quality of Life, *VAS* Visual analogue scale

^a^Self-reported pain, physical functioning in sports and recreation and quality of life measured by the Copenhagen Hip and Groin Outcome Score and physical functioning measured with the Short version of the International Hip Outcome Tool

^b^Exercise Adherence Rating Scale

^c^A senior lecturer, the first and fourth authors

## Methods

### Overarching research design

**Fig. 2:**
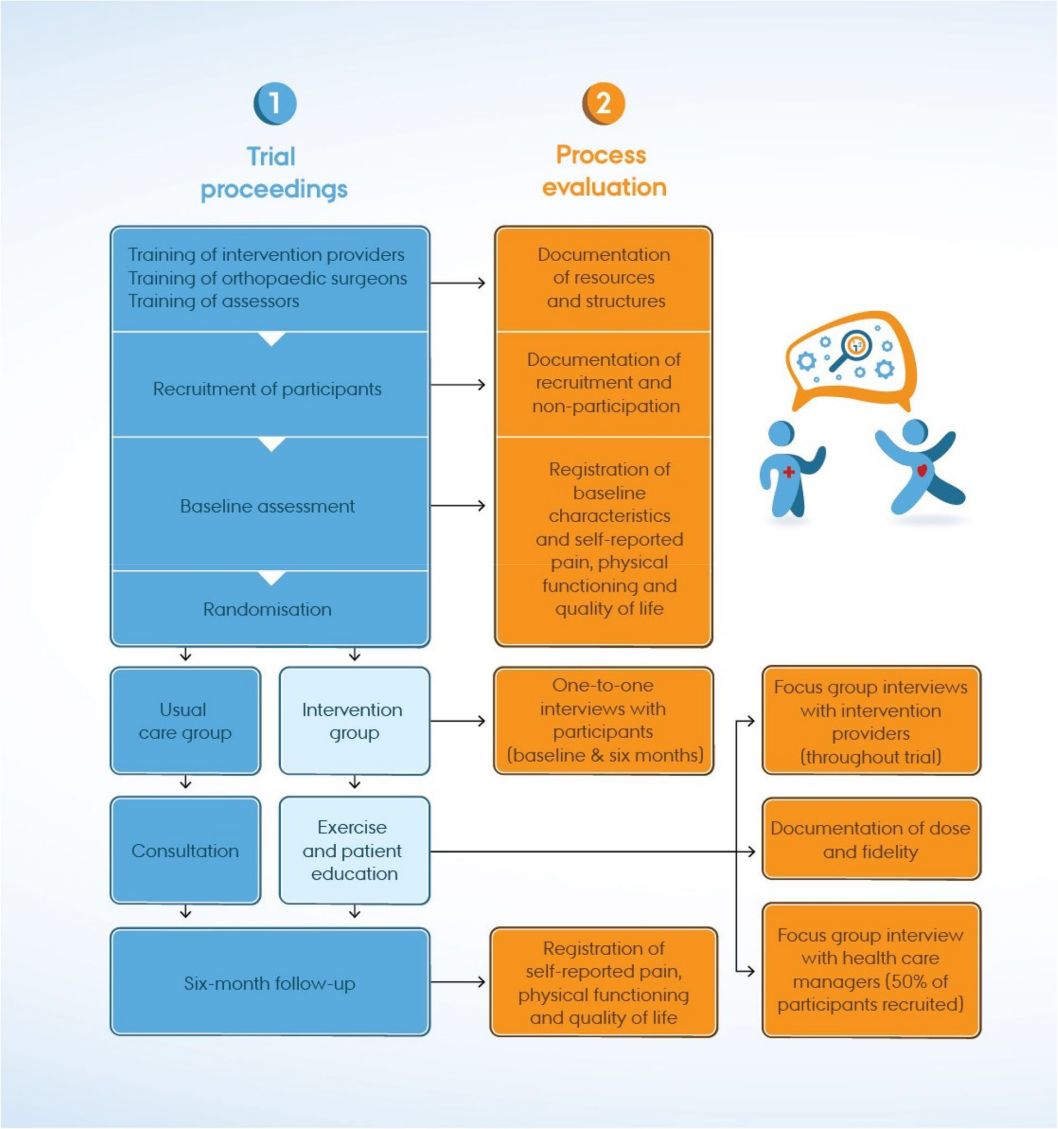
Flowchart of the MovetheHip trial proceedings and process evaluation. Abbreviations: SDT, self-determination theory; MI, motivational interviewing; HAGOS, Copenhagen Hip and Groin Outcome Score; iHOT-12, Short version of the International Hip Outcome Tool; PAO, periacetabular osteotomy

The overarching study design is a parallel-group superiority RCT with integrated process and health economic evaluation [21] designed in line with MRC Guidance principles [20]. The RCT follows the Standard Protocol Items: Recommendations for Interventional Trials statement [27] (Additional file 1). The process evaluation will follow a concurrent design with data collection and analyses being completed during similar time frames [28] (Fig. 2). The trial participants will be 18–50 years old and will have radiographically verified acetabular dysplasia and have experienced hip pain for a minimum of 3 months [21]. The participants will be dichotomised into either eligible but unwilling to undergo a PAO or not eligible for the PAO [21]. Further information on the study setting, eligibility criteria, sample size, etc., is reported in the RCT protocol [21]. In brief, to obtain sufficient statistical power, the RCT will recruit a minimum of 85 participants to the intervention group and 85 to the control group (usual care). Usual care includes one oral consultation provided by the first author on self-management of hip symptoms, including advice about staying physically active and exercising and, if relevant, advice to lose weight [21]. All intervention group participants will be included in the generation of process data. The RCT aims to investigate the effectiveness and cost-effectiveness of exercise and patient education compared with usual care [21].

### Implementation

We will conduct a multi-component examination of the implementation of the intervention, including process, fidelity, dose and reach [21]. We will examine the implementation process by describing the required structures and resources, and we will measure fidelity as the degree to which the intervention is delivered as intended. Dose will be measured as the number of completed supervised and home-based exercise sessions. High dose is defined as completing a minimum of 75% of scheduled training sessions, medium as completing 50–74% and low as completing less than 50% [21]. Additionally, we will measure the dose using the exercise adherence rating scale (EARS) [29, 30]. Reach will be examined by describing baseline participant characteristics in those receiving a high compared versus a low dose, and by comparing the age and sex of participants with those of non-participants. In an additional explorative analysis, we will examine if the dose is associated with changes in health outcomes.

### Acceptability of the intervention

We will explore acceptability by examining the extent to which the participants, intervention providers and healthcare managers consider the intervention to be appropriate based on their anticipated (prospective acceptability) or experienced responses to the intervention and received training (concurrent and retrospective acceptability) [31]. In an additional explorative analysis, we will explore if the reason for deselecting surgery (i.e. dichotomised into surgeon’s decision (not a surgical candidate) and participant’s decision (unwilling to undergo surgery)) moderates dose.

### Mechanisms of change

Mechanisms of change will be assessed by analysing how participants interact with the intervention activities to facilitate change in health outcomes. We will examine if autonomy, competence and relatedness relate to motivation and change in health outcomes and study the influence of contextual factors [22, 23].

### Contextual factors

Contextual factors include events and a socioecological view of personal, interpersonal and organisational factors interacting with the implementation, acceptability and mechanisms of change. By adopting a socioecological perspective, we will consider the implication of individual attitudes and behaviour and the quality of bonds to the intervention providers and support from family and friends. Furthermore, we will consider the impact of organisational support to gain additional knowledge on how processes may be optimised to facilitate any up-scaling of the intervention.

### Data sources

**Fig. 3:**
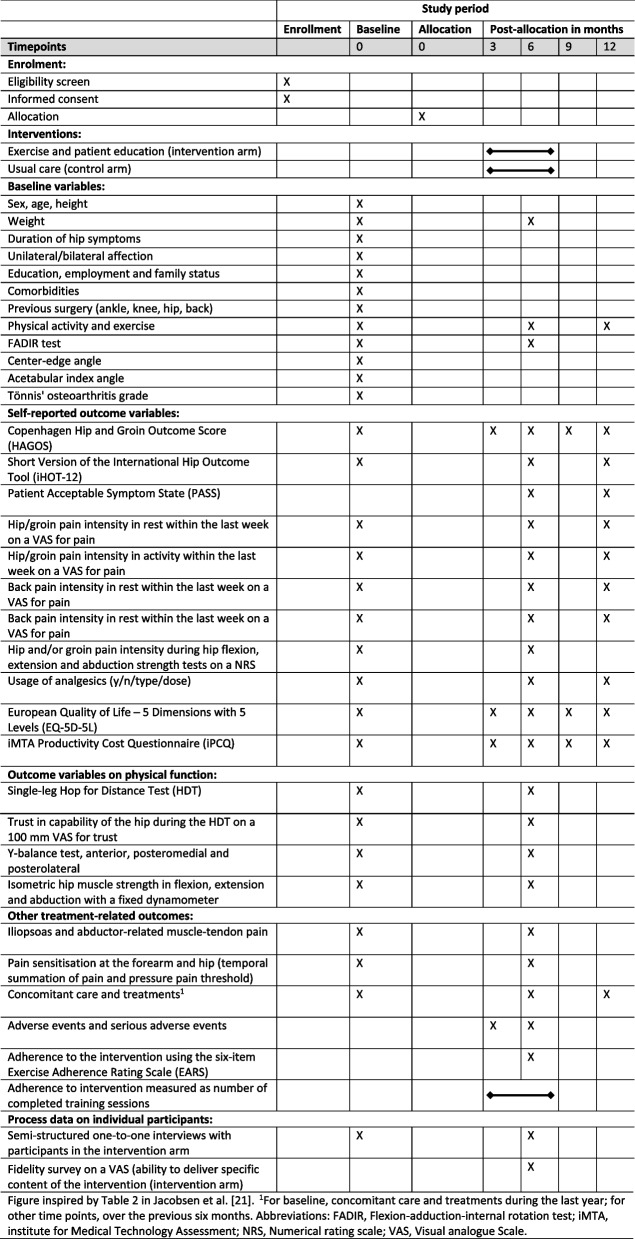
Schedule of procedures for the MovetheHip randomised controlled trial (SPIRIT figure)

The following sections outline data collection methods and sources relevant to the process evaluation. The timing and relation to the research questions are described in Table 1. Trial procedures are described in Fig. 3. The trial protocol paper details all wider measures obtained as part of the effectiveness trial [21].

#### Participant data measured

Orthopaedic surgeons and research assistants will measure and register baseline participant characteristics as reported in the trial protocol [21]. The baseline data will be used to describe intervention reach.

#### Online participant survey

Participants will enter baseline data using a survey option in a Research Electronic Data Capture (REDCap) database. Baseline process data include sex, age, duration of hip symptoms, educational level, employment status, cohabiting status and level of physical activity [21]. These data will be used to examine intervention reach as aforementioned.

#### Online participant-reported health outcome measures (health outcomes)

Self-reported pain, physical functioning in sports and recreation and quality of life will be measured using the Copenhagen Hip and Groin Outcome Score (HAGOS) [32]. Self-reported hip-related quality of life will be measured using the Short version of the International Hip Outcome Tool (iHOT-12) [33]. These health outcomes will be used to examine the underlying mechanisms of change by exploring how any changes in health outcomes are related to satisfaction of psychosocial needs and motivation.

#### Participant training records

Intervention dose is the number of completed training sessions (supervised and home-based) and the more comprehensive measure of doses received using the EARS [21]. The participants will prospectively register the number of completed training sessions using a weekly logbook and register with the EARS [29] the extent to which the four exercises described in the exercise programme are completed. The dose data will serve to examine intervention reach as aforementioned.

#### Online fidelity survey

Intervention providers will use a 100-mm visual analogue scale (VAS) in a REDCap survey form to measure their ability to deliver specific content of the intervention, ranging from not possible (0) to always possible (100). The intervention components describe the ability to deliver the following: (1) use the Borg CR10 to determine the difficulty level and repetitions of exercises; (2) use the participant’s expressions of exercise acceptability to determine difficulty level and repetition; (3) use the intervention manual to determine correct exercise performance; (4) council participants about pain mechanisms in acetabular dysplasia, give advice on physical activity, monitor weight loss (if relevant) and deliver support to increase exercise adherence. The data will be used to examine intervention tailoring as an integral aspect of the implementation process.

#### Semi-structured interviews with participants

Participants will be invited to participate in semi-structured one-to-one interviews by purposeful sampling at baseline and at the 6-month follow-up. Sampling will take into account factors such as gender, age, employment status and reason for not undergoing surgery. Data saturation will be checked prospectively, and further data collection will be undertaken if needed. The mode of the interviews will be either physical or via a video connection according to the participant’s preference. Baseline interviews will focus on previous experiences and expectations to changes and behaviour (degree of external, integrated or internal motivation) and the influence of personal, interpersonal and organisational contextual factors. The 6-month follow-up interviews will focus on experiences with the intervention, its acceptability and the participant’s behaviour (degree of external, integrated or internal motivation), as well as the influence of personal, interpersonal or organisational contextual factors. A semi-structured interview guide is developed and key questions are included in Table 2. As mentioned above, the interview data will be used to examine the underlying mechanisms of change.

**Table 2:** Process evaluation domains, themes and key questions for semi-structured one-to-one interviews with participants

Domains	Theme	Questions
***Baseline interview (pre-intervention delivery)***		
Prospective acceptability	Individual experiences	What are your experiences with exercising or being physically active?
	Expected contextual influences	What are your experiences about following a specific exercise programme?
	Individual expectations	What do you expect to gain from the participation?
Mechanisms of change	Individual expectations and beliefs about change, behaviour and motivation	What do you anticipate would inspire you to do exercises and physical activity?
	Expected contextual influences	What do you anticipate would stop you from exercising and doing physical activities?
***Six-month follow-up interview (post-intervention delivery)***		
Retrospective acceptability	Individual experiences	How would you describe your experience of being involved in the intervention?
		What did you enjoy most about the intervention?
		What did you find most challenging about the intervention?
		Which experiences do you keep from this intervention (sustainability)?
Mechanisms of change	Individual experiences	In what way has the intervention made a difference for you?
		You have received instructions from a personal instructor – What are your experiences with receiving individual instructions and getting feedback?
	Individual experiences and contextual influence	What encouraged you to get your exercises done?
		What prevented you from getting your exercises done?

#### Semi-structured focus group interviews with the intervention providers and the expert team

Regular evaluation meetings designed as online focus group interviews with the intervention providers and the expert team will be conducted in the study period [9, 21]. A semi-structured interview guide has been developed and key questions are included in Table 3. Focus group interviews will focus on the quality of delivery, comprising challenges, tailoring and experiences with the MovetheHip intervention. Data from these interviews will be used to examine how the intervention providers tailor the intervention to the individual participant, considering their different exercise behaviours and physical and mental functioning as part of the implementation examination. In addition, data will reflect a consensus about tailoring for the individual participant and will show how well the intervention providers feel prepared to deliver the intervention.

**Table 3:** Process evaluation domains, themes and key questions for interviews with intervention providers

Domain	Theme	Questions
*Participant behaviour*		
Concurrent acceptability	Exercises	Based on your observation of exercises, what challenges do the participants have when doing the exercises?
	Patient education	How do you experience that the participants comprehend your counselling about: - Acetabular dysplasia and pain mechanisms - Exercise and physical activity - Pain and overweight
Mechanisms of change	Motivation	How do you experience that the participants receive the incorporated flexibility of the intervention?
		How do you experience that the participants receive your feedback approach?
*Intervention provider behaviour*		
Concurrent acceptability	Exercises	What challenges do you have in instructing participants in the exercises?
	Patient education	What challenges do you experience when counselling about: - Acetabular dysplasia and pain mechanism? - Exercise and physical activity? - Pain and overweight?
Mechanisms of change	Motivation	How does it work for you to deliver the intervention with flexibility?
		How do you give feedback to the participants?
		How do you support or counsel the participants to integrate exercises and physical activity into everyday life?
		How do you approach counselling about acetabular dysplasia, pain, physical activity, etc.?
		What do you find is essential when you establish your relation to the participant?
*Participant tailoring*		
Implementation	Exercises	Based on your observation of exercises, how do you experience the participants’ ability to adjust the exercises to their performance ability?
	Patient education	How do you experience the participants' ability to translate counselling into actions in everyday life?
*Intervention provider tailoring*		
Implementation	Exercises	How do you adapt the exercises to the individual participant?
		Which adaptations did you make to instruct the participants on how to perform the exercises?
	Patient education	When delivering the education component to the participants, what considerations did you have when adapting your guidance to the individual participant?
*Intervention provider experiences*		
Concurrent acceptability	Experiences	What are your overall experiences with the intervention regarding what works and opportunities for improvement?
		How do you perceive your quality of delivery?
		What do you think about the training you received?
		Do you feel sufficiently prepared to deliver the intervention?
		Was the training you received acceptable in terms of: - What worked? - Have you missed anything? - How you have used the expert team?

#### Semi-structured focus group interview with healthcare managers and practice consultants

We will conduct an online semi-structured focus group interview with 4–6 key healthcare managers and practice consultants within the field of physiotherapy. The managers were public and private physical therapy clinic managers and lead managers in local municipalities responsible for health and rehabilitation resources. Furthermore, we will invite practice consultants within physiotherapy from the Central Denmark Region. A semi-structured interview guide has been developed and key questions are included in Table 4.

**Table 4:** Process evaluation domains, themes and key questions for an interview with healthcare managers

Domain	Theme	Questions
**Acceptability**	Experiences	What immediate thoughts do you have regarding the implementation of new interventions for citizens/patients in your organisation, or the organisations that you are involved with?
		What do you find most important when you decide or recommend if a new intervention should be implemented in your organisation, or the organisations that you are involved with?
		What strategies do you use when implementing or recommending new interventions to ensure that employees have the best prerequisites for following the new recommendations?
		What strategies do you use when implementing or recommending new interventions to ensure employees´ knowledge and skills?
	Priorities	How do you, in your organisation or the organisations you are involved with, decide if an intervention is relevant to implement?
		Do you, in your organisation or the organisations you are involved with, have any services where you can envision the MovetheHip intervention being added to the current treatment options?
**Implementation**	Context	Please tell me about a successful implementation
		Please tell me about a less successful implementation
	Knowledge	What knowledge about the MovetheHip intervention do you find most crucial to assess in terms of their relevance and benefits?
		- What type of evidence do you need?
	Context	What would support a successful implementation of the MovetheHip intervention?
		What would prevent a successful implementation of the MovetheHip intervention?

This focus group interview will explore intervention acceptability regarding how healthcare managers and consultants decide if an intervention is relevant and beneficial in their organisation; specifically, what information or evidence they need, and how they prioritise interventions and provide the necessary financial resources. Furthermore, the interview will explore their experiences with inter-organisational behaviour change. The data from the interviews with the healthcare managers may add to our understanding of what type of information healthcare managers consider essential. This understanding is important should they consider supporting a forthcoming implementation of this intervention, provided it proves to be effective and cost-effective.

### Quantitative data analysis

Descriptive statistics will be used on implementation data; normally distributed continuous data will be reported as means with standard deviations and categorical data will be reported as numbers and proportions. Mechanisms of change will be examined by linking intervention functioning (qualitative data) to mean changes in health outcomes. Specifically, mean changes in health outcomes will be calculated in normally distributed data using descriptive statistics. Furthermore, we will explore the possibility of dichotomising data on intervention dose into low versus high. Reach will be examined by describing baseline characteristics within each dose group using descriptive statistics.

Additional explorative analyses will be performed. To examine the implementation, we will explore if the dose is associated with changes in health outcomes, using simple linear regression models with doses as independent variables and changes in health outcomes as dependent variables. Furthermore, in a repeated measurement analysis using a mixed-effects model, an explorative analysis will be undertaken to determine if the reason for deselecting surgery (i.e. dichotomised into the surgeon’s or participant’s decision) moderates dose changes as part of the examination of acceptability. In the model, participants will be the random effects with a fixed factor for group and time and the corresponding interaction (group × time), adjusted for baseline values. Statistical significance is considered to have been achieved at *p* < 0.05, and the Stata 17 (StataCorp, College Station, TX, USA) software package is used for the data analyses.

### Qualitative data analysis

All interviews will be recorded and transcribed verbatim. A theoretical thematic analysis will be conducted [34]. Blinded to the findings of the RCT, the last author (DS) will index a subset of the data and construct a coding framework for each dataset (i.e. individual interviews and focus group interviews). A priori codes covering the process evaluation domains and concepts from the self-determination theory and motivational interviewing will be included in the coding framework, and these domains and concepts will be supplemented with new codes emerging from the data. The remaining data will be analysed according to the analytic framework. The last author will code the first interviews of each dataset and refine each coding framework. Subsequently, the first author (JSJ) will code the remaining data according to the coding framework. The last author will then verify all coded data and refine them if needed. Once all data have been coded, the last author will identify relevant themes within each dataset. Themes from across all datasets will be compared and refined to agree on a final set of study-level themes. These themes will be accompanied by anonymised quotes, again collected by the last author.

### Data integration

Data integration will be achieved through data merging once the qualitative and quantitative data have been collected and analysed separately [28]. The analysis focuses on the following three process evaluation domains: the degree to which the intervention is implemented, the acceptability, and how the intervention functions (mechanisms of change). Furthermore, the analysis will explore how contextual factors relate to these three process evaluation domains. Integration at the interpretation and reporting level will follow the weaving approach, involving writing the qualitative and quantitative findings together on a theme-by-theme basis according to the process evaluation domains and research questions [28]. The findings made during data integration will show if the quantitative and qualitative findings confirm, expand or are in discordance with each other [28].

## Discussion

The present protocol paper presents a detailed protocol for the process evaluation of the 6-month exercise and patient education intervention of the MovetheHip trial [21]. The process evaluation will determine how the intervention functions, and the findings from the evaluation will be used to refine the programme theory and enhance our understanding of how the theory-informed MovetheHip intervention relates to motivation and observed management of pain and physical limitations in people with acetabular dysplasia. Finally, if the intervention is proven effective, process data will determine how the intervention may be optimised to facilitate its future up-scaling and implementation.

The process evaluation will draw upon the strengths of both quantitative and qualitative data by integrating data at the reporting level through the weaving approach [28]. This presents an opportunity to comprehend the perspectives of those interviewed and integrate them with the quantitative data [28]. For example, quotes regarding the functioning of the intervention can be incorporated into the data concerning changes in health outcomes. This integration can be applied across each process evaluation domain and is considered a strength of the evaluation.

The implementation of the intervention may face various challenges, including adherence to the intervention and maintaining fidelity. Some participants may find it challenging to maintain the required training dose, particularly when tasked with repeating four exercises several times a week [35]. Additionally, contextual factors such as family and work-related responsibilities could further hinder exercise adherence [35]. Similarly, delivering the intervention with fidelity may be complicated by the heterogeneous nature of the population, which encompasses diverse needs and aspirations, potentially influencing what can be effectively delivered [8, 9]. Nevertheless, in the current process evaluation, we will consider the expectations and experiences of both participants and intervention providers, with the possibility of integrating findings across these groups. These perspectives of participants and intervention providers have the potential to enhance the understanding of what can be delivered.

We choose physiotherapy students as intervention providers to make the intervention implementable in any location, setting and context. Most public and private clinics that treat people with acetabular dysplasia employ both experienced and less experienced physiotherapists, and the skills of the lesser-trained physiotherapists would probably be similar to those of physiotherapy students. Thus, most clinics will be able to adapt our training approach without needing special courses or education.

## Trial status

As of January 2024, at the time of writing, 76 out of the required 200 participants have been enrolled since data collection commenced in April 2021. Data collection is planned to conclude by July 2026. Protocol version: 01 and date: 17 Jan 2024.

### Supplementary Information

Additional file 1. Spirit checklist. A completed Spirit checklist

## Data Availability

The first author has access to the final trial dataset and is responsible for communication trial results to participants, healthcare professionals, the public and policy makers through peer-reviewed scientific journals and conferences, seminars and through social media. The public will have access to the present protocol and the RCT protocol through publication in open access journals. Public access to participants-level data will not be given.

## Abbreviations

HAGOS: Copenhagen Hip and Groin Outcome Score
EARS: Exercise Adherence Rating Scale
PAO: Periacetabular osteotomy
RCT: Randomised controlled trial
REDCap: Research Electronic Data Capture
iHOT-12: Short version of the International Hip Outcome Tool
VAS: Visual analogue scale

## References

[1] Mechlenburg I, Nyengaard JR, Rømer L, Søballe K (2004). Changes in load-bearing area after Ganz periacetabular osteotomy evaluated by multislice CT scanning and stereology. Acta Orthop Scand.

[2] Larsen JB, Mechlenburg I, Jakobsen SS, Thilleman TM, Søballe K (2020). 14-year hip survivorship after periacetabular osteotomy: a follow-up study on 1,385 hips. Acta Orthop.

[3] Clohisy JC, Ackerman J, Baca G, Baty J, Beaule PE, Kim YJ (2017). Patient-reported outcomes of periacetabular osteotomy from the prospective ANCHOR cohort study. J Bone Joint Surg - Am.

[4] O’Brien MJM, Jacobsen JS, Semciw AI, Mechlenburg I, Tønning LU, Stewart CJW (2022). Physical impairments in Adults with Developmental Dysplasia of the Hip (DDH) undergoing Periacetabular osteotomy (PAO): a systematic review and meta-analysis. Int J Sports Phys Ther.

[5] Coobs BR, Xiong A, Clohisy JC (2015). Contemporary concepts in the young adult hip patient: Periacetabular osteotomy for hip dysplasia. J Arthroplasty.

[6] Novais EN, Potter GD, Clohisy JC, Millis MB, Kim YJ, Trousdale RT (2015). Obesity is a major risk factor for the development of complications after peri-acetabular osteotomy. Bone Joint J..

[7] Jakobsen SS, Overgaard S, Søballe K, Ovesen O, Mygind-Klavsen B, Dippmann CA (2018). The interface between periacetabular osteotomy, hip arthroscopy and total hip arthroplasty in the young adult hip. EFORT Open Rev.

[8] Jorgensen MD, Frederiksen SB, Sørensen D, Jacobsen JS (2021). Experiences of living with developmental dysplasia of the hip in adults not eligible for surgical treatment: a qualitative study. BMJ Open.

[9] Jacobsen JS, Thorborg K, Sørensen D, Jakobsen SS, Nielsen RO, Ostergaard LG (2022). Feasibility and acceptability of a six-month exercise and patient education intervention for patients with hip dysplasia: A mixed methods study. Musculoskelet Sci Pract.

[10] Harris-Hayes M, Czuppon S, Van Dillen LR, Steger-May K, Sahrmann S, Schootman M (2016). Movement-pattern training to improve function in people with chronic hip joint pain: a feasibility randomized clinical trial. J Orthop Sports Phys Ther.

[11] Kuroda D, Maeyama A, Naito M, Moriyama S, Yoshimura I, Nakamura Y (2013). Dynamic hip stability, strength and pain before and after hip abductor strengthening exercises for patients with dysplastic hips. Isokinet Exerc Sci.

[12] Mortensen L, Schultz J, Elsner A, Jakobsen S, Søballe K, Jacobsen J (2018). Progressive resistance training in patients with hip dysplasia: a feasibility study. J Rehabil Med.

[13] Miller LS, Gramzow RH (2016). A self-determination theory and motivational interviewing intervention to decrease racial/ethnic disparities in physical activity: rationale and design. BMC Public Health.

[14] Ryan RM, Deci EL (2000). Self-determination theory and the facilitation of intrinsic motivation, social development, and well-being. Am Psycho.

[15] Deci EL, Ryan RM (2000). The ‘what’ and ‘why’ of goal pursuits: Human needs and the self-determination of behavior. Psychol Inq.

[16] Miller WR, Rollnich S (2013). Motivational Interviewing: Helping People Change.

[17] Fortier MS, Sweet SN, O’Sullivan TL, Williams GC (2007). A self-determination process model of physical activity adoption in the context of a randomized controlled trial. Psychol Sport Exerc.

[18] Silva MN, Vieira PN, Coutinho SR, Minderico CS, Matos MG, Sardinha LB (2010). Using self-determination theory to promote physical activity and weight control: A randomized controlled trial in women. J Behav Med.

[19] Van Hoecke AS, Delecluse C, Opdenacker J, Lipkens L, Martien S, Boen F (2013). Long-term effectiveness and mediators of a need-supportive physical activity coaching among Flemish sedentary employees. Health Promot Int.

[20] Skivington K, Matthews L, Simpson SA, Craig P, Baird J, Blazeby JM (2021). A new framework for developing and evaluating complex interventions: update of Medical Research Council guidance. BMJ.

[21] Jacobsen JS, Thorborg K, Nielsen RØ, Jakobsen SS, Foldager C, Sørensen D (2022). Comparing exercise and patient education with usual care in the treatment of hip dysplasia: a protocol for a randomised controlled trial with 6-month follow-up (MovetheHip trial). BMJ Open.

[22] Moore G, Audrey S, Barker M, Bond L, Bonell C, Hardeman W, et al. Process Evaluation of Complex Interventions Guidance: UK Medical Research Council (MRC) Guidance. MRC Population Health Sciences Research Network; 2014. Available from: https://www.ukri.org/publications/process-evaluation-of-complexinterventions/. Cited 2024 Jun 26.

[23] Moore GF, Audrey S, Barker M, Bond L, Bonell C, Hardeman W (2015). Process evaluation of complex interventions: Medical Research Council guidance. BMJ (Online).

[24] Ntoumanis N, Ng JYY, Prestwich A, Quested E, Hancox JE, Thøgersen-Ntoumani C (2021). A meta-analysis of self-determination theory-informed intervention studies in the health domain: effects on motivation, health behavior, physical, and psychological health. Health Psychol Rev.

[25] Borg GA (1982). Psychophysical bases of perceived exertion. Med Sci Sports Exerc.

[26] Deci EL, Ryan RM. The ‘What’ and ‘Why’ of Goal Pursuits: Human Needs and the Self-Determination of Behavior. Psychol Inq. 2000;11:227–68.

[27] Chan AW, Tetzlaff JM, Gøtzsche PC, Altman DG, Mann H, Berlin JA, SPIRIT,  (2013). explanation and elaboration: guidance for protocols of clinical trials. BMJ.

[28] Fetters MD, Curry LA, Creswell JW (2013). Achieving Integration in Mixed Methods Designs—Principles and Practices. Health Serv Res.

[29] Jacobsen JS, Nielsen RO, Godfrey EL (2022). Translation and Cross-Cultural Adaptation of the Exercise Adherence Rating Scale (EARS) into Danish. Transl Sports Med.

[30] Newman-Beinart NA, Norton S, Dowling D, Gavriloff D, Vari C, Weinman JA (2017). The development and initial psychometric evaluation of a measure assessing adherence to prescribed exercise: the Exercise Adherence Rating Scale (EARS). Physiotherapy.

[31] Sekhon M, Cartwright M, Francis JJ (2017). Acceptability of healthcare interventions: an overview of reviews and development of a theoretical framework. BMC Health Serv Res.

[32] Thorborg K, Hölmich P, Christensen R, Petersen J, Roos EM (2011). The Copenhagen Hip and Groin Outcome Score (HAGOS): development and validation according to the COSMIN checklist. Br J Sports Med.

[33] Griffin DR, Parsons N, Mohtadi NGH, Safran MR (2012). A short version of the International Hip Outcome Tool (iHOT-12) for use in routine clinical practice. Arthroscopy.

[34] Braun V, Clarke V (2006). Using thematic analysis in psychology. Qual Res Psychol.

[35] Collado-Mateo D, Lavín-Pérez AM, Peñacoba C, Del Coso J, Leyton-Román M, Luque-Casado A (2021). Key factors associated with adherence to physical exercise in patients with chronic diseases and older adults: an umbrella review. Int J Environ Res Public Health..

