# Supplementary material for: An exercise and patient education intervention to reduce pain and physical limitations in adults with acetabular dysplasia: study protocol for a process evaluation integrated within a randomised controlled trial (the MovetheHip trial)
Source: Trials. 2024 Jun 24;25:411. doi: 10.1186/s13063-024-08262-y (PMC11197205; doi:10.1186/s13063-024-08262-y)
Supplement: Supplementary file 1 — Additional file 1. Spirit checklist. A completed Spirit checklist [file 13063_2024_8262_MOESM1_ESM.pdf]

SPIRIT Checklist for *Trials*

Complete this checklist by entering the page and line numbers where each of the items listed below can be found in your manuscript.

Your manuscript may not currently address all the items on the checklist. Please modify your text to include the missing information. If you are certain that an item does not apply, please state "n/a" and provide a short explanation. **Leaving an item blank or stating "n/a" without an explanation will lead to your manuscript being returned before review.**

Upload your completed checklist as an additional file when you submit to *Trials*. You must reference this additional file in the main text of your protocol submission. The completed SPIRIT figure must be included within the main body of the protocol text and can be downloaded here: http://www.spirit-statement.org/schedule-of-enrolment-interventions-and-assessments/

In your methods section, please state that you used the SPIRIT reporting guidelines, and cite them as:

Chan A-W, Tetzlaff JM, Gøtzsche PC, Altman DG, Mann H, Berlin J, Dickersin K, Hróbjartsson A, Schulz KF, Parulekar WR, Krleža-Jerić K, Laupacis A, Moher D. SPIRIT 2013 Explanation and Elaboration: Guidance for protocols of clinical trials. BMJ. 2013;346:e7586

		**Reporting Item**	**Page and Line Number**	**Reason if not applicable**
**Administrative information**				
Title	#1	Descriptive title identifying the study design, population, interventions, and, if applicable, trial acronym	1 (1-3)	
Trial registration	#2a	Trial identifier and registry name. If not yet registered, name of intended registry	4 (75-78	
Trial registration: data set	#2b	All items from the World Health Organization Trial Registration Data Set	20 (418-423)	
Protocol version	#3	Date and version identifier	19 (409-411)	

Funding	#4	Sources and types of financial, material, and other support	20 (435-442)	
Roles and responsibilities: contributorship	#5a	Names, affiliations, and roles of protocol contributors	1-2 (6-29), 21 (443-458)	
Roles and responsibilities: sponsor contact information	#5b	Name and contact information for the trial sponsor	1 (14-15), 2 (28-29)	
Roles and responsibilities: sponsor and funder	#5c	Role of study sponsor and funders, if any, in study design; collection, management, analysis, and interpretation of data; writing of the report; and the decision to submit the report for publication, including whether they will have ultimate authority over any of these activities	20 (440-442)	
Roles and responsibilities: committees	#5d	Composition, roles, and responsibilities of the coordinating centre, steering committee, endpoint adjudication committee, data management team, and other individuals or groups overseeing the trial, if applicable (see Item 21a for data monitoring committee)	20 (435-442)	
**Introduction**				
Background and rationale	#6a	Description of research question and justification for undertaking the trial, including summary of relevant studies (published and	5 (93-112), 8-9 (187-199)	

		unpublished) examining benefits and harms for each intervention		
Background and rationale: choice of comparators	#6b	Explanation for choice of comparators	9 (211-213)	Additional details in trial protocol: https://bmjopen.bmj.com/content/12/9/e064242
Objectives	#7	Specific objectives or hypotheses	8-9 (187-199)	
Trial design	#8	Description of trial design including type of trial (eg, parallel group, crossover, factorial, single group), allocation ratio, and framework (eg, superiority, equivalence, non-inferiority, exploratory)	9 (201-211)	Additional details in trial protocol: https://bmjopen.bmj.com/content/12/9/e064242
**Methods: Participants, interventions, and outcomes**				
Study setting	#9	Description of study settings (eg, community clinic, academic hospital) and list of countries where data will be collected. Reference to where list of study sites can be obtained	7-8 (152-180)	Additional details in trial protocol: https://bmjopen.bmj.com/content/12/9/e064242
Eligibility criteria	#10	Inclusion and exclusion criteria for participants. If applicable, eligibility criteria for study centres and individuals who will perform the interventions (eg, surgeons, psychotherapists)	9 (206-210), 8 (165-180)	Additional details in trial protocol: https://bmjopen.bmj.com/content/12/9/e064242
Interventions: description	#11a	Interventions for each group with sufficient detail to allow replication, including how and when they will be administered	5-7 (113-151), 9 (211-213)	Additional details in trial protocol: https://bmjopen.bmj.com/content/12/9/e064242
Interventions: modifications	#11b	Criteria for discontinuing or modifying allocated interventions for a given trial	n/a	The present protocol describes a process evaluation whereas details about discontinuing

		participant (eg, drug dose change in response to harms, participant request, or improving / worsening disease)		etc. are reported in the trial protocol: https://bmjopen.bmj.com/content/12/9/e064242
Interventions: adherance	#11c	Strategies to improve adherence to intervention protocols, and any procedures for monitoring adherence (eg, drug tablet return; laboratory tests)	n/a	The present protocol describes a process evaluation whereas details about strategies to improve adherence etc. are reported in the trial protocol: https://bmjopen.bmj.com/content/12/9/e064242
Interventions: concomitant care	#11d	Relevant concomitant care and interventions that are permitted or prohibited during the trial	n/a	The present protocol describes a process evaluation whereas details about concomitant care etc. are reported in the trial protocol: https://bmjopen.bmj.com/content/12/9/e064242
Outcomes	#12	Primary, secondary, and other outcomes, including the specific measurement variable (eg, systolic blood pressure), analysis metric (eg, change from baseline, final value, time to event), method of aggregation (eg, median, proportion), and time point for each outcome. Explanation of the clinical relevance of chosen efficacy and harm outcomes is strongly recommended	12-16 (261-334), Fig. 3 page 31-32.	Additional details in trial protocol: https://bmjopen.bmj.com/content/12/9/e064242
Participant timeline	#13	Time schedule of enrolment, interventions (including any run-ins and washouts), assessments, and visits for participants. A schematic diagram is highly recommended (see Figure)	n/a	The present protocol describes a process evaluation whereas details about enrolment etc. are reported in the trial protocol: https://bmjopen.bmj.com/content/12/9/e064242

Sample size	#14	Estimated number of participants needed to achieve study objectives and how it was determined, including clinical and statistical assumptions supporting any sample size calculations	9 (210-211)	Additional details in trial protocol: https://bmjopen.bmj.com/content/12/9/e064242
Recruitment	#15	Strategies for achieving adequate participant enrolment to reach target sample size	n/a	The present protocol describes a process evaluation whereas details about strategies for achieving adequate participant enrolment are reported in the trial protocol: https://bmjopen.bmj.com/content/12/9/e064242
**Methods: Assignment of interventions (for controlled trials)**				
Allocation: sequence generation	#16a	Method of generating the allocation sequence (eg, computer-generated random numbers), and list of any factors for stratification. To reduce predictability of a random sequence, details of any planned restriction (eg, blocking) should be provided in a separate document that is unavailable to those who enrol participants or assign interventions	n/a	The present protocol describes a process evaluation whereas details about randomisation and allocation are reported in the trial protocol: https://bmjopen.bmj.com/content/12/9/e064242
Allocation concealment mechanism	#16b	Mechanism of implementing the allocation sequence (eg, central telephone; sequentially numbered, opaque, sealed envelopes), describing any steps to conceal the sequence until interventions are assigned	n/a	The present protocol describes a process evaluation whereas details about randomisation and allocation are reported in the trial protocol: https://bmjopen.bmj.com/content/12/9/e064242
Allocation: implementation	#16c	Who will generate the allocation sequence, who will enrol participants, and who will assign participants to interventions	n/a	The present protocol describes a process evaluation whereas details about randomisation

				and allocation are reported in the trial protocol: https://bmjopen.bmj.com/content/12/9/e064242
Blinding (masking)	#17a	Who will be blinded after assignment to interventions (eg, trial participants, care providers, outcome assessors, data analysts), and how	n/a	The present protocol describes a process evaluation whereas details about blinding are reported in the trial protocol: https://bmjopen.bmj.com/content/12/9/e064242
Blinding (masking): emergency unblinding	#17b	If blinded, circumstances under which unblinding is permissible, and procedure for revealing a participant's allocated intervention during the trial	n/a	The present protocol describes a process evaluation whereas details about blinding are reported in the trial protocol: https://bmjopen.bmj.com/content/12/9/e064242
**Methods: Data collection, management, and analysis**				
Data collection plan	#18a	Plans for assessment and collection of outcome, baseline, and other trial data, including any related processes to promote data quality (eg, duplicate measurements, training of assessors) and a description of study instruments (eg, questionnaires, laboratory tests) along with their reliability and validity, if known. Reference to where data collection forms can be found, if not in the protocol	12-16 (261-334), Fig. 3 page 31-32.	Additional details in trial protocol: https://bmjopen.bmj.com/content/12/9/e064242
Data collection plan: retention	#18b	Plans to promote participant retention and complete follow-up, including list of any outcome data to be collected for participants who discontinue or deviate from intervention protocols	n/a	The present protocol describes a process evaluation whereas details to promote retention are reported in the trial protocol: https://bmjopen.bmj.com/content/12/9/e064242

Data management	#19	Plans for data entry, coding, security, and storage, including any related processes to promote data quality (eg, double data entry; range checks for data values). Reference to where details of data management procedures can be found, if not in the protocol	n/a	The present protocol describes a process evaluation whereas details about data entry etc. are reported in the trial protocol: https://bmjopen.bmj.com/content/12/9/e064242
Statistics: outcomes	#20a	Statistical methods for analysing primary and secondary outcomes. Reference to where other details of the statistical analysis plan can be found, if not in the protocol	16 (335-353)	Additional details in trial protocol: https://bmjopen.bmj.com/content/12/9/e064242
Statistics: additional analyses	#20b	Methods for any additional analyses (eg, subgroup and adjusted analyses)	17 (354-367), 18 (368-378)	Additional details in trial protocol: https://bmjopen.bmj.com/content/12/9/e064242
Statistics: analysis population and missing data	#20c	Definition of analysis population relating to protocol non-adherence (eg, as randomised analysis), and any statistical methods to handle missing data (eg, multiple imputation)	n/a	The present protocol describes a process evaluation whereas details about analysis population related to protocol non-adherence etc. are reported in the trial protocol: https://bmjopen.bmj.com/content/12/9/e064242
**Methods: Monitoring**				
Data monitoring: formal committee	#21a	Composition of data monitoring committee (DMC); summary of its role and reporting structure; statement of whether it is independent from the sponsor and competing interests; and reference to where further details about its charter can be found, if not in the protocol. Alternatively, an explanation of why a DMC is not needed	n/a	The present protocol describes a process evaluation whereas details about data monitoring are reported in the trial protocol: https://bmjopen.bmj.com/content/12/9/e064242

Data monitoring: interim analysis	#21b	Description of any interim analyses and stopping guidelines, including who will have access to these interim results and make the final decision to terminate the trial	n/a	No interim analysis is planned for the trial
Harms	#22	Plans for collecting, assessing, reporting, and managing solicited and spontaneously reported adverse events and other unintended effects of trial interventions or trial conduct	n/a	The present protocol describes a process evaluation whereas details about adverse events are reported in the trial protocol: https://bmjopen.bmj.com/content/12/9/e064242
Auditing	#23	Frequency and procedures for auditing trial conduct, if any, and whether the process will be independent from investigators and the sponsor	21 (447-450)	
**Ethics and dissemination**				
Research ethics approval	#24	Plans for seeking research ethics committee / institutional review board (REC / IRB) approval	20 (418-423)	
Protocol amendments	#25	Plans for communicating important protocol modifications (eg, changes to eligibility criteria, outcomes, analyses) to relevant parties (eg, investigators, REC / IRBs, trial participants, trial registries, journals, regulators)	20 (426-431)	
Consent or assent	#26a	Who will obtain informed consent or assent from potential trial participants or authorised surrogates, and how (see Item 32)	n/a	The present protocol describes a process evaluation whereas details about who obtain informed consent are reported in the trial protocol: https://bmjopen.bmj.com/content/12/9/e064242

Consent or assent: ancillary studies	#26b	Additional consent provisions for collection and use of participant data and biological specimens in ancillary studies, if applicable	n/a	Data is not used in ancillary studies
Confidentiality	#27	How personal information about potential and enrolled participants will be collected, shared, and maintained in order to protect confidentiality before, during, and after the trial	n/a	The present protocol describes a process evaluation whereas details about how personal information is collected etc. are reported in the trial protocol: https://bmjopen.bmj.com/content/12/9/e064242
Declaration of interests	#28	Financial and other competing interests for principal investigators for the overall trial and each study site	20 (433-334)	Additional details in trial protocol: https://bmjopen.bmj.com/content/12/9/e064242
Data access	#29	Statement of who will have access to the final trial dataset, and disclosure of contractual agreements that limit such access for investigators	20 (426-431)	Additional details in trial protocol: https://bmjopen.bmj.com/content/12/9/e064242
Ancillary and post trial care	#30	Provisions, if any, for ancillary and post-trial care, and for compensation to those who suffer harm from trial participation	n/a	The present protocol describes a process evaluation whereas details about provisions etc. are reported in the trial protocol: https://bmjopen.bmj.com/content/12/9/e064242
Dissemination policy: trial results	#31a	Plans for investigators and sponsor to communicate trial results to participants, healthcare professionals, the public, and other relevant groups (eg, via publication, reporting in results databases, or other data sharing arrangements), including any publication restrictions	20 (426-431)	

Dissemination policy: authorship	#31b	Authorship eligibility guidelines and any intended use of professional writers	n/a	The present protocol describes a process evaluation whereas details about authorship etc. are reported in the trial protocol: https://bmjopen.bmj.com/content/12/9/e064242
Dissemination policy: reproducible research	#31c	Plans, if any, for granting public access to the full protocol, participant-level dataset, and statistical code	20 (426-431)	
**Appendices**				
Informed consent materials	#32	Model consent form and other related documentation given to participants and authorised surrogates	n/a	The present protocol descibes a process evaluation whereas details about consent etc. are reported in the trial protocol: https://bmjopen.bmj.com/content/12/9/e064242
Biological specimens	#33	Plans for collection, laboratory evaluation, and storage of biological specimens for genetic or molecular analysis in the current trial and for future use in ancillary studies, if applicable	n/a	The trial does not involve biological material.

It is strongly recommended that this checklist be read in conjunction with the SPIRIT 2013 Explanation & Elaboration for important clarification on the items. Amendments to the protocol should be tracked and dated. The SPIRIT checklist is copyrighted by the SPIRIT Group under the Creative Commons "Attribution-NonCommercial-NoDerivs 3.0 Unported" license. This checklist can be completed online using https://www.goodreports.org/, a tool made by the EQUATOR Network in collaboration with Penelope.ai
